# Cantharidin and norcantharidin inhibit the ability of MCF-7 cells to adhere to platelets via protein kinase C pathway-dependent downregulation of α2 integrin

**DOI:** 10.3892/or.2013.2601

**Published:** 2013-07-08

**Authors:** LIU-MEI SHOU, QIONG-YAN ZHANG, WEI LI, XIN XIE, KAI CHEN, LIAN LIAN, ZHEN-YU LI, FEI-RAN GONG, KE-SHENG DAI, YI-XIANG MAO, MIN TAO

**Affiliations:** 1 Department of Oncology, The First Affiliated Hospital of Soochow University, Suzhou, Jiangsu 215006, P.R. China; 2 Department of Hematology, The First Affiliated Hospital of Soochow University, Suzhou, Jiangsu 215006, P.R. China; 3 Jiangsu Institute of Hematology, The First Affiliated Hospital of Soochow University, Suzhou, Jiangsu 215006, P.R. China; 4 Key Laboratory of Thrombosis and Hemostasis of Ministry of Health, The First Affiliated Hospital of Soochow University, Suzhou, Jiangsu 215006, P.R. China; 5 Department of Oncology, Suzhou Xiangcheng People’s Hospital, Suzhou, Jiangsu 215131, P.R. China; 6 Jiangsu Institute of Clinical Immunology, Suzhou, Jiangsu 215006, P.R. China; 7 Department of Molecular and Cellular Biochemistry, University of Kentucky, Lexington, KY 40536, USA

**Keywords:** cantharidin and norcantharidin, breast cancer, platelet, integrin α2, protein kinase C, protein phosphatase 2A

## Abstract

Cancer metastasis is a highly coordinated and dynamic multistep process in which cancer cells interact with a variety of host cells. Morphological studies have documented the association of circulating tumor cells with host platelets, where a surface coating of platelets protects tumor cells from mechanical trauma and the immune system. Cantharidin is an active constituent of mylabris, a traditional Chinese medicine. Cantharidin and norcantharidin are potent protein phosphatase 2A (PP2A) inhibitors that exhibit *in vitro* and *in vivo* antitumor activity against several types of cancer, including breast cancer. We investigated whether cantharidin and norcantharidin could repress the ability of MCF-7 breast cancer cells to adhere to platelets. Using MTT, clone formation, apoptosis, adhesion and wound-healing assays, we found that cantharidin and norcantharidin induced apoptosis and repressed MCF-7 cell growth, adhesion and migration. Moreover, we developed a flow cytometry-based analysis of tumor cell adhesion to platelets. We proved that cantharidin and norcantharidin repressed MCF-7 cell adhesion to platelets through downregulation of α2 integrin, an adhesion molecule present on the surface of cancer cells. The repression of α2 integrin expression was found to be executed through the protein kinase C pathway, the activation of which could have been due to PP2A inhibition.

## Introduction

Breast cancer is the most frequent cause of mortality in women in the developed world. Although early detection, precise resection using wide margins, and systematic adjuvant therapy have improved survival, distant metastasis remains the leading cause of breast cancer-related mortality (1). Circulating tumor cells (CTCs) are isolated tumor cells that disseminate from the sites of metastatic and/or primary cancer, including breast cancer. We have proved that CTCs can be identified and measured in the peripheral blood (1). The rate of detection and number of CTCs correlate with the disease stage in breast cancer patients. Moreover, the assessment of CTCs in metastatic breast cancer patients could predict the efficacy of chemotherapy (1). It is generally accepted that tumor cells are damaged by the immune system and physical factors (i.e., shear force and mechanical trauma due to passage through the microvasculature) during circulatory transport. However, when covered with platelets, cancer cells can overcome these stresses in the blood stream. The involvement of platelets in hematogenous metastasis has long been recognized. Recent studies have shown that the risk of cancer diagnosis is elevated after primary deep vein thrombosis or pulmonary embolism (2). Thus, therapies targeting the interaction between cancer cells and platelets could be a promising strategy to prevent cancer metastasis.

In previous studies, cantharidin and its derivatives exhibited strong *in vitro* and *in vivo* antitumor activity against the cells of various types of cancer (3–5), including breast cancer (6). In our previous studies, we found that cantharidin repressed cancer cell growth through cell cycle arrest and the induction of apoptosis (7–9). In the present study, we investigated the effect of cantharidin and norcantharidin on the ability of metastatic human breast cancer MCF-7 cells to adhere to platelets. The mechanism involved was also investigated.

## Materials and methods

### Cell culture

MCF-7 human breast cancer cells from the American Type Culture Collection (ATCC, Manassas, VA, USA) were maintained in RPMI-1640 (Gibco, Grand Island, NY, USA) supplemented with 10% fetal calf serum (HyClone, Logan, UT, USA), 100 U/ml penicillin, and 100 mg/ml streptomycin at 37°C in a humidified atmosphere containing 5% CO_2_. The cells were passaged every two to three days to maintain exponential growth.

### Reagents

Cantharidin, PD98059, SP600125, GF109203X and Ro 31-8220 were purchased from Enzo Life Sciences International Inc., (Plymouth Meeting, PA, USA). Norcantharidin was purchased from Sigma (St. Louis, MO, USA).

### MTT assay

Cellular growth was evaluated by the MTT (3-[4,5-dimethylthiazol-2-yl]-2,5-diphenyltetrazolium bromide) assay (10). The cells were seeded into 24-well tissue culture plates at 5×10^4^ cells/well. Following treatment, MTT (Sigma) was added to each well at a final concentration of 0.5 mg/ml, followed by incubation at 37°C for 4 h. The medium was then removed and 800 μl dimethyl sulfoxide was added to each well. The absorbance of the mixture was measured at 490 nm using a microplate ELISA reader (Bio-Rad Laboratories, Hercules, CA, USA). The inhibition rate was calculated as follows: inhibition rate = [(mean control absorbance − mean experimental absorbance)/mean control absorbance] × 100%. To evaluate the effect of cantharidin and norcantharidin on cellular growth, the concentration that caused 50% growth inhibition (IC_50_) was calculated, as previously described (7).

### Plate clone formation assay

The cells were seeded at a density of 200 cells/well in 24-well plates and treated 12 h later. After 15 days, the cells were stained with 1% methylrosanilinium chloride and the numbers of visible colonies were counted. The relative clone formation ability was calculated as relative clone formation ability = (mean experimental clone number/mean control clone number) × 100%.

### Apoptosis assays

Apoptosis was evaluated using the Annexin V-FITC/PI Apoptosis Detection kit (Biouniquer Technology, Nanjing, China) according to the manufacturer’s instructions. The cells were resuspended in binding buffer, and Annexin V-FITC and propidium iodide (PI) were added to the buffer and incubated at room temperature for 15 min in the dark, followed by flow cytometry using a Beckman Coulter FC500 dual-laser five-color flow cytometer (Beckman Coulter, Fullerton, CA, USA).

### Adhesion assay

The cells were resuspended in complete medium and seeded in 24-well plates at a concentration of 1×10^4^ cells/ml. After a 5-h incubation, the unattached cells were removed to another well. The attached cells and unattached cells were evaluated using the MTT assay. The adhesion rate was calculated as: [absorbance of attached cells/(absorbance of attached cells + absorbance of unattached cells)] × 100%.

### Wound healing assay

The cells were seeded in 96-well plates at a density of 1×10^4^ cells/well and grown to confluence. The monolayer culture was then artificially scrape-wounded with a sterile micropipette tip to create a denuded zone (gap) of constant width. Each well was washed with phosphate-buffered saline (PBS) twice to remove the detached cells before treatment. Cells that had migrated to the wounded region were observed using an XDS-1B inverted microscope (Chongqing Optical & Electrical Instrument Co., Ltd., Chongqing, China) and photographed (x40 magnification). Images were captured every 4 h to monitor the wound healing process. The wound areas were measured using ImageJ (National Institutes of Health, Bethesda, MA, USA).

### Platelet preparation and fluorescence labeling

Fresh blood obtained from healthy volunteers was anticoagulated with a 1/7 volume of acid-citrate dextrose (85 mM trisodium citrate, 110 mM dextrose and 78 mM citric acid) as previously described (11). The Ethics Committee of the First Affiliated Hospital of Soochow University, Suzhou, China, approved the study. Platelet-rich plasma was collected after centrifugation at 1,300 rpm for 13 min. Then, CFDA-SE (carboxyfluorescein diacetate succinimidyl ester; Beyotime, Shanghai, China) was added at a final concentration of 5 μM and incubated at room temperature for 10 min. The fluorescence-labeled platelets were then washed twice with CGS buffer (0.12 M sodium chloride, 0.0129 M trisodium citrate and 0.03 M D-glucose, pH 6.5), resuspended in freshly prepared Tyrode’s buffer (11), and allowed to rest for at least 1 h at 37°C prior to use.

### Analysis of tumor cell adhesion to platelets

The traditional platelet adhesion assay was performed as previously described (12). In brief, 5×10^4^ cells/ml MCF-7 cells were seeded in a 96-well plate and grown until confluent. Washed platelets preloaded with CFDA-SE were allowed to adhere to the cells for 30 min at 37°C. The non-adherent platelets were discarded. Platelet adhesion to the MCF-7 cells was imaged by Olympus IX51 inverted fluorescence microscopy (Olympus, Tokyo, Japan). The flow cytometry-based platelet adhesion assay was performed as follows: the cells were harvested and resuspended in RPMI-1640 containing 1 mM CaCl_2_, 1 mM MgCl_2_ and 0.1% bovine serum albumin. One hundred microliters of cells (5×10^6^ cells/ml) were incubated with 400 μl CFDA-SE-labeled platelets for 30 min. Then, 4% paraformaldehyde (250 μl) was added to a final concentration of 2%. The cells were fixed for 1 h at 4°C, followed by flow cytometry analysis.

### Real-time PCR

Total RNA was extracted using TRIzol reagent (Invitrogen, Carlsbad, CA, USA) according to the manufacturer’s protocol. After spectrophotometric quantification, 1 μg total RNA was used for reverse transcription in a final volume of 20 μl with AMV reverse transcriptase (Promega, Madison, WI, USA) according to the manufacturer’s instructions. Aliquots cDNA of corresponding to equal amounts of RNA were used for the quantification of mRNA by real-time PCR using the TL988-II Real-time Quantitative PCR Detection system (TianLong Science And Technology, Co., Ltd., Shaanxi, China). The reaction system (25 μl) contained the corresponding cDNA, forward and reverse primers, and SYBR Green PCR Master Mix (Roche Diagnostics, Indianapolis, IN, USA). All data were analyzed using β-actin gene expression as an internal standard. The specific primers were: i) integrin α2, forward, 5′-CTGGAGTGGCTTTCCTGAG-3′ and reverse, 5′-ACTG ATTCCCACATTGCTG-3′, product, 224 bp; ii) integrin β1, forward, 5′-GAGATGGGAAACTTGGTGG-3′ and reverse, 5′-GACAAGGTGAGCAATAGAAGGAT-3′, product, 113 bp; iii) p65, forward, 5′-GAGCCGCACAGCATTCAGG-3′ and reverse, 5′-CGCTGCATCCACAGTTTCCA-3′, product, 156 bp; and iv) β-actin, forward, 5′-TCATGAAGTGTGA CGTGGACAT-3′ and reverse, 5′-CTCAGGAGGAGCAA TGATCTTG-3′, product, 158 bp.

### Abundance of α2 integrin on the cell surface

The abundance of α2 integrin on the surface of the MCF-7 cells was measured by flow cytometry. Following treatment, the cells were incubated with saturating concentrations (10 μg/ml) of anti-α2 integrin antibody clone P1E6 for 30 min at room temperature. Subsequently, the cells were incubated with 10 μg/ml fluorescein-isothiocyanate-conjugated anti-mouse antibody for 30 min at room temperature and were analyzed using a Beckman Coulter FC500 dual-laser five-color flow cytometer (Beckman Coulter).

### Transfection of small interfering RNA

Target-specific small interfering RNAs (siRNAs) were designed and synthesized by GenePharma (Shanghai, China). The specific sequences were as: i) control-siRNA, sense, 5′-UUCUCCGAACGUG UCACGUdTdT-3′ and antisense, 5′-ACGUGACACGUUCG GAGAAdTdT-3′; ii) p65-siRNA-1, sense, 5′-CGGAUUGAG GAGAAACGUAdTdT-3′ and antisense, 5′-UACGUUUCUC CUCAAU CCGdTdT-3′; iii) p65-siRNA-2, sense, 5′-GGAGU ACCCUGAGGCUAUAdTdT-3′ and antisense, 5′-UAUAGC CUCAGGGUACUCCdTdT-3′. The transfections were performed with the siRNA-Mate Transfection reagent (GenePharma) according to the manufacturer’s instructions.

### Statistical analysis

Each experiment was performed in triplicate, at minimum. The results are expressed as the means ± standard deviation. Statistical analysis was performed using unpaired Student’s t-tests; P<0.05 was considered to indicate a statistically significant different.

## Results

### Cantharidin and norcantharidin exert multiple inhibitory effects on the biological behaviors of MCF-7 cells

**Figure 1:**
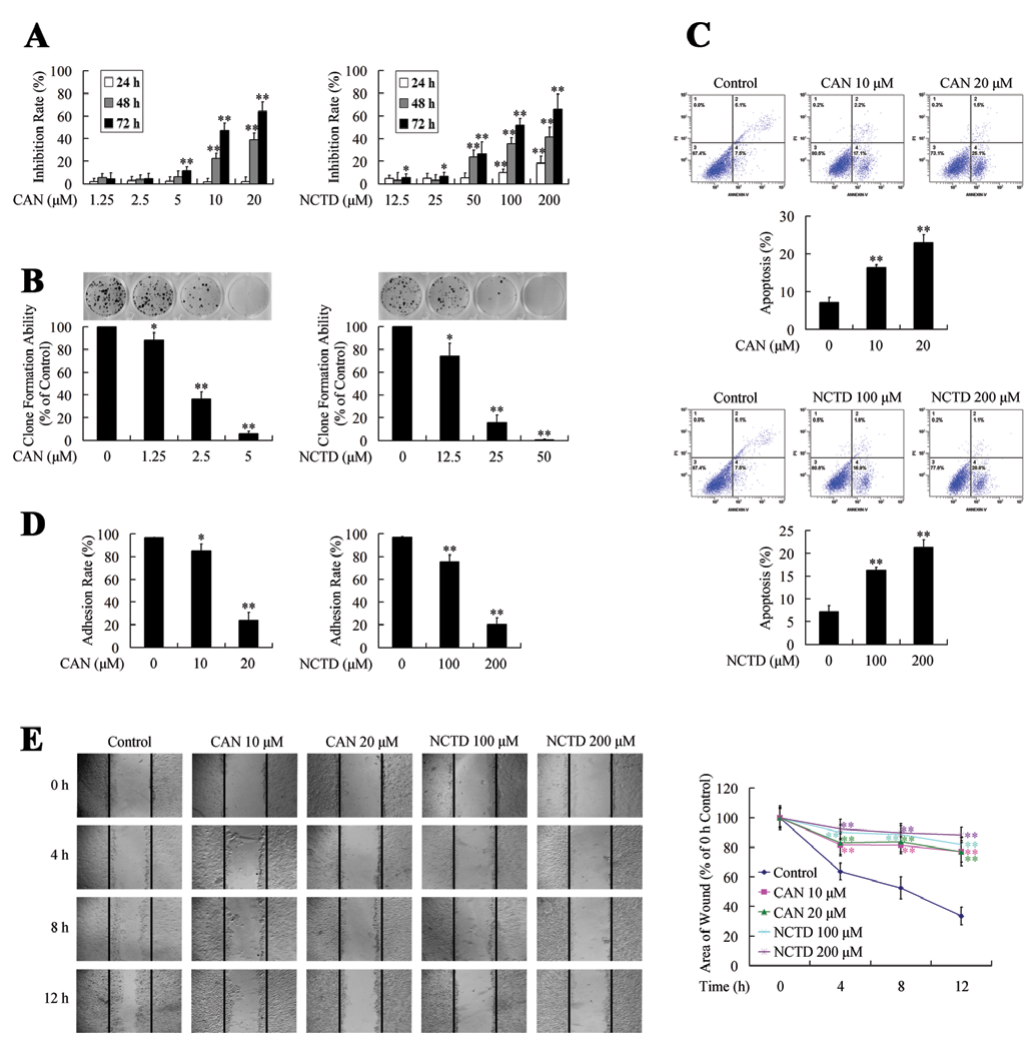
Inhibitory effect of cantharidin (CAN) and norcantharidin (NCTD) on the biological behavior of MCF-7 cells. (A) Exposure to various concentrations of CAN and NCTD resulted in dose- and time-dependent growth inhibition. (B) CAN and NCTD treatment inhibited the clone formation ability of MCF-7 cells in a dose-dependent manner. (C) Following CAN and NCTD treatment, the early apoptotic (Annexin V+/PI−) cell populations increased significantly in a dose-dependent manner. (D) CAN and NCTD treatment inhibited MCF-7 cell adhesion in a dose-dependent manner. (E) CAN and NCTD treatment inhibited MCF-7 cell migration in a time-dependent manner. ^*^P<0.05; ^**^P<0.01 indicate significant differences from the respective control groups.

First, we investigated the effects of cantharidin and norcantharidin on the growth, adhesion and migration of the MCF-7 cells. The inhibitory effects of cantharidin and norcantharidin on MCF-7 cell growth were first evaluated using the MTT assay. As presented in Fig. 1A, cantharidin or norcantharidin treatment inhibited MCF-7 cell growth in a dose- and time-dependent manner, and the IC_50_ values at 72 h after treatment were 11.96 and 105.34 μM, respectively. The dose-dependent inhibitory effect of cantharidin and norcantharidin on the growth of MCF-7 cells was also confirmed by clone-formation assays (Fig. 1B).

We performed apoptosis assays to further investigate the mechanism involved in the growth inhibition effect. The percentages of cell populations at various stages of apoptosis are shown in Fig. 1C. After cantharidin or norcantharidin treatment, the number of cells that underwent early apoptosis (Annexin V+/PI−) increased significantly in a dose-dependent manner. These data suggested that the growth-inhibition effect of cantharidin and norcantharidin could be due to induction of apoptosis.

The effects of cantharidin and norcantharidin on the adhesion and migration ability of MCF-7 cells were also evaluated. Cantharidin or norcantharidin dose-dependently repressed the adhesion ability of MCF-7 cells (Fig. 1D) and repressed cell migration in a dose- and time-dependent manner (Fig. 1E).

Thus, the above data indicate inhibition of multiple biological behaviors of MCF-7 cells following cantharidin and norcantharidin treatment.

### Cantharidin and norcantharidin inhibit the adhesion of MCF-7 cells to platelets

**Figure 2:**
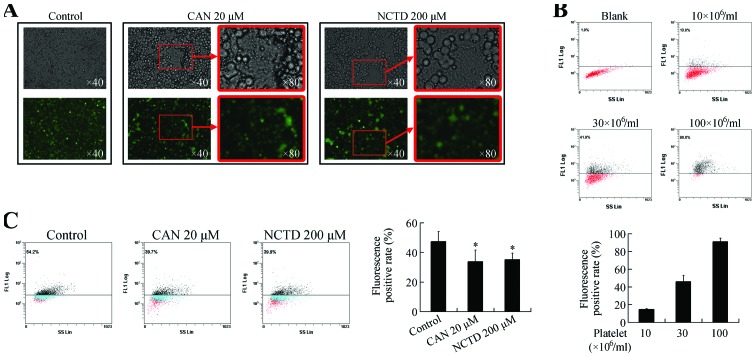
(A) Photomicrographs and fluorescence microscopy images of adhesion between MCF-7 cells and platelets. (B) Flow cytometry-based platelet adhesion assay. The fluorescent positive rate increased when the platelet concentration increased. (C) Cantharidin (CAN) and norcantharidin (NCTD) treatment decreased the fluorescent positive rate. ^*^P<0.05 vs. control group.

We then focused on the effect of cantharidin and norcantharidin on the adhesion of MCF-7 cells to platelets. First, we observed the appearance of the cells following 24-h cantharidin and norcantharidin treatment. As shown in Fig. 2A, the untreated cells were confluent, whereas several gaps were observed in the cantharidin or norcantharidin-treated groups. This could have been due to the decreased cell number induced by growth inhibition, as well as the detachment triggered by the decreased adhesion ability and apoptosis as presented in Fig. 1. Thus, when the traditional platelet adhesion assay (12) was performed, the platelets adhered directly to the plate through the gaps (Fig. 2A). Therefore, the fluorescent intensity did not accurately reflect the actual ability of the MCF-7 cells to adhere the platelets.

Consequently, we developed a platelet adhesion assay based on the flow cytometry. As shown in Fig. 2B, the fluorescent positive rate was increased when the dose of platelets was increased. When incubated with 10×10^6^/ml platelets, few cells were covered by the platelets, while the adhesion became saturated when the platelet concentration was increased to 100×10^6^ cells/ml. Thus, a moderate concentration of 30×10^6^ cells/ml was used for further investigation.

As shown in Fig. 2C, the fluorescent rate was repressed when the cells were treated with cantharidin or norcantharidin, suggesting that cantharidin and norcantharidin could inhibit the adhesion between the MCF-7 cells and platelets. Thus, anticancer therapy using cantharidin and norcantharidin might be able to repress the platelet-mediated hematogenous metastasis potential of breast cancer cells.

### Cantharidin and norcantharidin inhibit the ability of MCF-7 cells to adhere to platelets through downregulation of α2 integrin

α2β1 integrin, a heterodimeric transmembrane receptor composed of the α2 and β1 subunits, is expressed by some tumor cells to facilitate binding to the extracellular matrix. Recent studies have suggested that this adhesion molecule could be involved in the interaction between cancer cells and platelets (13). Therefore, we investigated whether the regulation of α2β1 integrin expression could facilitate the inhibition of cancer cell-platelet adhesion induced by cantharidin and norcantharidin treatment.

**Figure 3:**
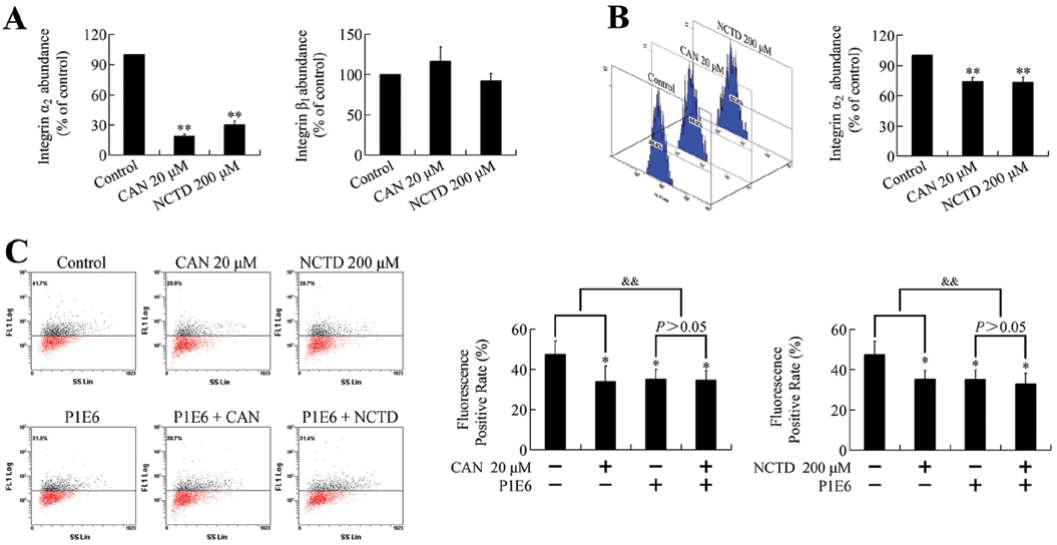
Cantharidin (CAN) and norcantharidin (NCTD) inhibit the ability of MCF-7 cells to adhere to platelets through the downregulation of α2 integrin. (A) CAN and NCTD downregulated the mRNA expression of α2 integrin, but had no effect on β1 integrin. (B) CAN and NCTD downregulated the abundance of α2 integrin on the surface of MCF-7 cells. (C) P1E6 repressed the adhesion between MCF-7 cells and platelets, and attenuated the repressed adhesion induced by CAN and NCTD. ^*^P<0.05; ^**^P<0.01 indicate significant differences from the respective control groups. ^&^P<0.01 indicates significant differences between fold inductions.

As presented in Fig. 3A, the mRNA expression of α2 integrin was downregulated following treatment with cantharidin or norcantharidin, while the expression of β1 integrin was not affected. The abundance of α2 integrin on the cell surface was further evaluated using flow cytometry. As shown in Fig. 3B, the distribution of α2 integrin on the cell surface was decreased upon cantharidin or norcantharidin treatment.

As cantharidin and norcantharidin treatment repressed the expression of α2 integrin, we subsequently investigated whether the inhibition of MCF-7 cell adhesion to platelets was executed due to downregulation of α2 integrin. As presented in Fig. 3C, P1E6, the function-blocking antibody to α2 integrin, repressed the adhesion between the MCF-7 cells and platelets, suggesting that α2 integrin mediated the interaction between the MCF-7 cells and the platelets. Moreover, pretreatment with P1E6 repressed the decline of the cantharidin and norcantharidin-treated MCF-7 cell adhesion to platelets, suggesting that cantharidin and norcantharidin repress the adhesion of MCF-7 cells to platelets by downregulating α2 integrin.

### Cantharidin and norcantharidin repress α2 integrin expression through the PKC pathway

**Figure 4:**
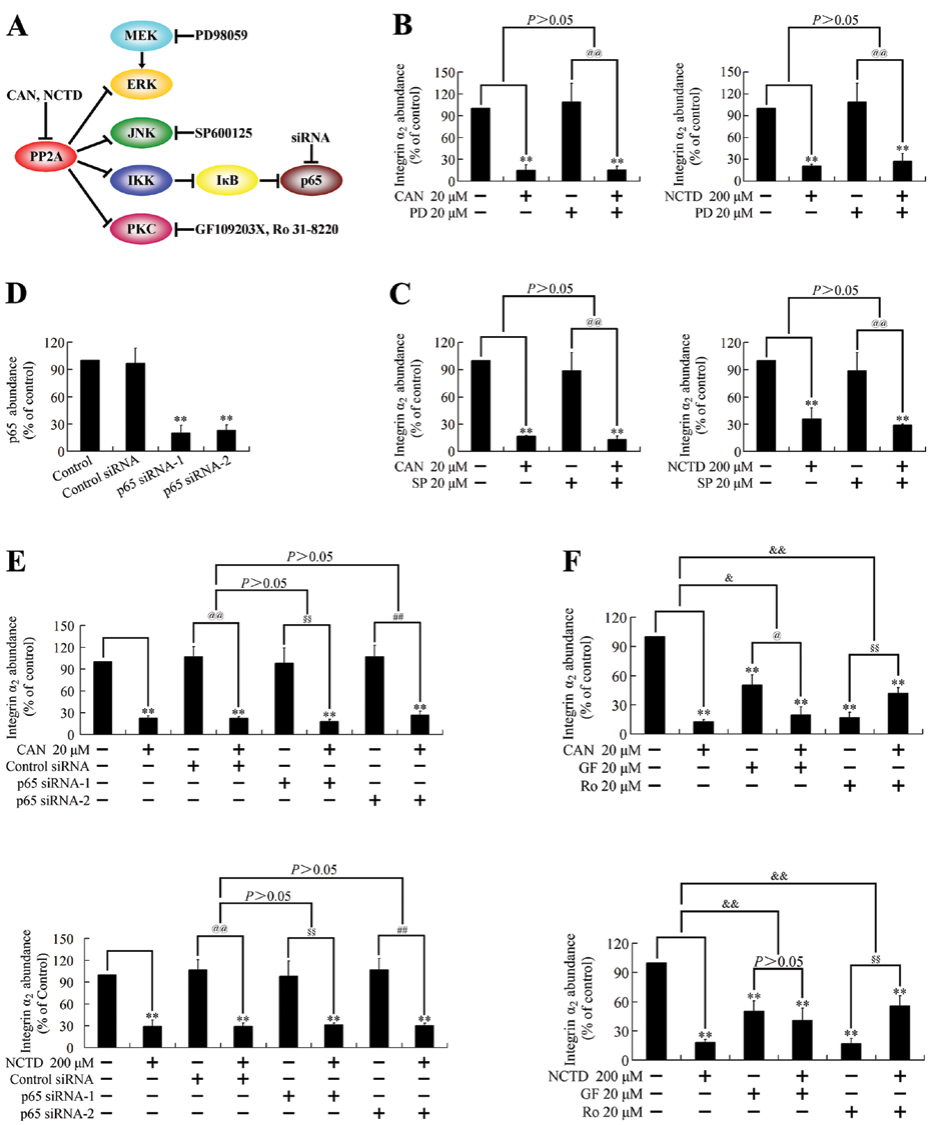
Mechanisms involved in the repression of α2 integrin expression by cantharidin (CAN) and norcantharidin (NCTD). (A) PP2A-regulated signaling pathways and the blockers of each pathway. (B) The effect of PD98059 (PD) on CAN and NCTD-induced α2 integrin downregulation. ^**^P<0.01 indicates significant differences from the respective control groups. ^@@^P<0.01 vs. PD group. (C) The effect of SP600125 (SP) on CAN and NCTD-induced α2 integrin downregulation. ^**^P<0.01 indicates significant differences from the respective control groups. ^@@^P<0.01 vs. SP group. (D) Knockdown of p65 using target-specific siRNA. ^**^P<0.01 indicates significant differences from the respective control groups. (E) Effect of p65-siRNA on CAN and NCTD-induced α2 integrin downregulation. ^**^P<0.01 indicates significant differences from the respective control groups. ^@@^P<0.01 vs. control-siRNA group. ^§§^P<0.01 vs. p65-siRNA-1 group. ^##^P<0.01 vs. p65-siRNA-2 group. (F) Effect of PKC inhibitors GF109203X (GF) and Ro 31-8220 (Ro) on CAN and NCTD-induced α2 integrin downregulation. ^**^P<0.01 indicates significant differences from the respective control groups. ^@^P<0.05 vs. GF group. ^§§^P<0.01 vs. Ro group. ^&^P<0.05; ^&^P<0.01 indicates significant differences between fold inductions.

Mechanistically, cantharidin and norcantharidin have been shown to be potent and selective inhibitors of protein phosphatase 2A (PP2A) (14), a multimeric serine/threonine phosphatase that can dephosphorylate several kinases (15,16). In our previous studies (7–9), we found that extracellular signal-regulated kinase (ERK), c-Jun N-terminal kinase (JNK), IκB kinase (IKK) and protein kinase C (PKC) were activated following treatment with PP2A inhibitors. To investigate whether these pathways were involved in the downregulation of α2 integrin, we tested the expression of α2 integrin following cantharidin and norcantharidin treatment when these pathways were blocked (Fig. 4A).

Pretreatment with PD98059, an ERK pathway inhibitor, or SP600125, a JNK pathway inhibitor, did not affect the downregulation of α2 integrin in cantharidin or norcantharidin-treated cells (Fig. 4B and C). Blocking the nuclear factor κB (NF-κB) by transfection of a siRNA-targeting p65 (Fig. 4D) did not affect the downregulation of α2 integrin either (Fig. 4E). However, pretreatment with the PKC inhibitors GF109203X or Ro 31-8220 attenuated the downregulation of α2 integrin (Fig. 4F). Ro 31-8220 even reversed the effects of cantharidin and norcantharidin on α2 integrin expression, indicating that cantharidin and norcantharidin repress α2 integrin expression through the PKC pathway.

## Discussion

The anticancer activity of cantharidin and norcantharidin has been explored in several studies (3–6). Previously, we found that cantharidin repressed pancreatic cancer cell growth by triggering apoptosis (7,8). In the present study, induction of apoptosis was also observed in MCF-7 cells treated with cantharidin and norcantharidin. Although other groups have studied the growth inhibition effect of cantharidin and norcantharidin on breast cancer cells (6), the effects on other biological behaviors have not been investigated. The present study found that cantharidin and norcantharidin treatment also inhibited the adhesion and migration abilities of MCF-7 cells. Moreover, we discovered for the first time that cantharidin and norcantharidin could repress cancer cell adhesion to platelets, which could repress the metastatic potential of the cells.

The adhesion analysis for tumor cells and platelets widely used at present can be divided into two approaches: i) the cells are seeded onto culture plates. After the cells are adhered, isotope or fluorescence-labeled platelets are added into the plates. Following incubation, unattached platelets are removed and the adhesion analysis is performed by detecting the radioactivity or fluorescence in the plates (12,17–19); or ii) platelets are seeded into culture plates and allowed to adhere. Then, the non-adherent platelets are removed and cancer cells are added into the plates. After incubation, unattached cells are removed and the attached cells are counted (20,21). As the cells or platelets are adhesive, these methods cannot mimic the physiological state in which both cells and platelets are suspended.

However, for the present study, the number of cantharidin and norcantharidin-treated cells was inhomogeneous to that of the control group, as the growth was repressed and the cells detached due to apoptosis and the repressed adhesion. Moreover, the platelets could directly adhere to the plates. Thus, we developed a flow cytometry-based quantitative adhesion analysis for tumor cells and platelets. Both the cancer cells and platelets were suspended during the test, mimicking the physiological state. With this method, we found that as the platelet concentration increased, more cancer cells were covered with platelets. This result was in agreement with the clinical observation that an increased platelet level indicates poorer cancer prognosis (2,13). In addition, this method permitted the novel observation that cantharidin and norcantharidin could repress cancer cell adhesion to platelets, which could repress their metastatic potential.

It is believed that α2β1 integrin is involved in the interaction of cancer cells and platelets (13). This interaction is thought to take place through collagen (13). However, the present study found that the α2 integrin expressed on the surface of the cancer cells mediated direct interaction between the cancer cells and platelets.

The downregulation of α2 integrin is a possible mechanism by which cantharidin and norcantharidin can repress cancer cell adhesion to platelets. It has been proven that the NF-κB and ERK cell-signaling pathways are involved in the transcriptional regulation of α2 integrin expression (22,23), suggesting that the downregulation of α2 integrin following cantharidin and norcantharidin treatment might involve these pathways. Mechanistically, cantharidin and norcantharidin have been proven to be PP2A inhibitors (14), which can induce the phosphorylation and activation of several substrate kinases, including ERK, JNK, PKC and IKK, the key regulator of the NF-κB pathway (15,16). Previously (7–9), we found that these pathways were activated following treatment with PP2A inhibitors. ERK activation had no effect on cell growth (7), while JNK activation repressed cell proliferation (7,9) and IKK activation triggered apoptosis (8). However, none of these pathways were found to participate in the downregulation of α2 integrin. Although the activation of PKC reduced the cytotoxic effect of cantharidin (9), activation of PKC was found to be responsible for the downregulation of α2 integrin in the present study. Thus, PKC activation fulfills different functions in different aspects of cell biological behaviors. Although both PKC inhibitors repressed the downregulation of α2 integrin, the inhibitors induced different magnitudes of effects. This could be due to the variant affinity of these two inhibitors to the multiple isoforms of PKC.

As PP2A can induce the phosphorylation and activation of several substrate kinases, most of which can accelerate tumor growth (15,16,24), PP2A inhibition has always been considered to be cancer promoting. However, in our previous and present studies, we proved that PP2A inhibitor-induced kinase activation repressed cancer cell proliferation, induced apoptosis and reduced the metastatic potential, suggesting that these putative cancer inducers are actually potential anticancer drugs.

In conclusion, in the present study, we investigated the anticancer effects of cantharidin and norcantharidin against MCF-7 breast cancer cells. Cantharidin and norcantharidin repressed the growth, adhesion, and migration abilities of MCF-7 cells. By developing a flow cytometry-based quantitative analysis of tumor cell adhesion to platelets, we discovered for the first time that cantharidin and norcantharidin could repress cancer cell adhesion to platelets, which could repress the metastatic potential. Further investigation of the mechanism demonstrated that cantharidin and norcantharidin repressed cancer cell adhesion to platelets through PKC-dependent downregulation of the expression of α2 integrin, an adhesion molecule on the surface of cancer cells.

## Abbreviations

CFDA-SE: carboxyfluorescein diacetate succinimidyl ester
CGS buffer: sodium chloride, trisodium citrate, D-glucose buffer
CTC: circulating tumor cell
ERK: extracellular signal-regulated kinase
IC_50_: 50% growth inhibition
IKK: IκB kinase
JNK: c-Jun N-terminal kinase
MTT: 3-[4,5-dimethylthiazol-2-yl]-2,5-diphenyltetrazolium bromide
NF-κB: nuclear factor κB
PBS: phosphate-buffered saline
PKC: protein kinase C
PP2A: protein phosphatase 2A
siRNAs: small interfering RNAs
